# Supplementary material for: Combining Pharmacokinetics and Vibrational Spectroscopy: MCR-ALS Hard-and-Soft Modelling of Drug Uptake In Vitro Using Tailored Kinetic Constraints
Source: Cells. 2022 May 5;11(9):1555. doi: 10.3390/cells11091555 (PMC9099467; doi:10.3390/cells11091555)
Supplement: Supplementary file 1 [file cells-11-01555-s001.zip › cells-1680078-supplementary.pdf]

Supplementary Material

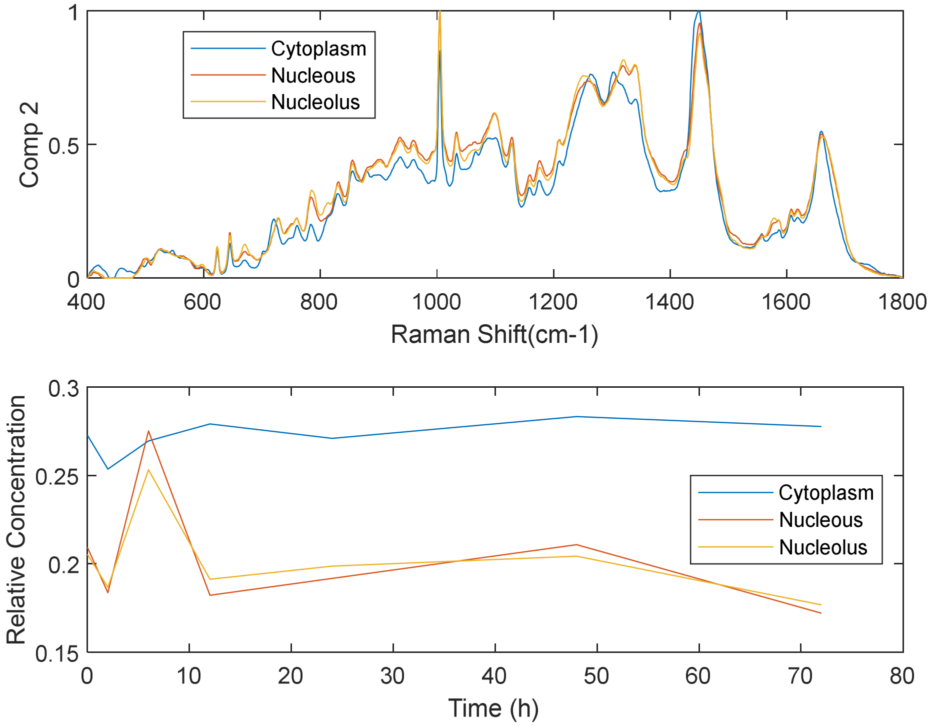

Supplementary Figure S1. Comparison of the pure spectra and concetration matrix calculated in section 3.1
